# Verapamil Ameliorates Motor Neuron Degeneration and Improves Lifespan in the SOD1^G93A^ Mouse Model of ALS by Enhancing Autophagic Flux

**DOI:** 10.14336/AD.2019.0228

**Published:** 2019-12-01

**Authors:** Xiaojie Zhang, Sheng Chen, Kaili Lu, Feng Wang, Jiangshan Deng, Zhouwei Xu, Xiuzhe Wang, Qinming Zhou, Weidong Le, Yuwu Zhao

**Affiliations:** ^1^Department of Neurology, Shanghai Jiaotong University Affiliated Sixth People's Hospital, Shanghai, China.; ^2^Department of Neurology, Ruijin Hospital, Shanghai Jiaotong University School of Medicine, Shanghai, China.; ^3^Liaoning Provincial Center for Clinical Research on Neurological Diseases, the First Affiliated Hospital, Dalian Medical University, Dalian, China.; ^4^Liaoning Provincial Kay Laboratory for Research on the Pathogenic Mechanisms of Neurological Diseases, the First Affiliated Hospital, Dalian Medical University, Dalian, China.; ^5^Collaborative Innovation Center for Brain Science, the First Affiliated Hospital, Dalian Medical University, Dalian, China.

**Keywords:** Amyotrophic lateral sclerosis, autophagy, verapamil, calpain 1, neurodegeneration

## Abstract

Amyotrophic lateral sclerosis (ALS) is a progressive, paralytic disorder caused by selective degeneration of motor neurons in the brain and spinal cord. Our previous studies indicated that abnormal protein aggregation and dysfunctional autophagic flux might contribute to the disease pathogenesis. In this study, we have detected the role of the Ca^2+^ dependent autophagic pathway in ALS by using the L-type channel Ca^2+^ blocker, verapamil. We have found that verapamil significantly delayed disease onset, prolonged the lifespan and extended disease duration in SOD1^G93A^ mice. Furthermore, verapamil administration rescued motor neuron survival and ameliorated skeletal muscle denervation in SOD1^G93A^ mice. More interestingly, verapamil significantly reduced SOD1 aggregation and improved autophagic flux, which might be mediated the inhibition of calpain 1 activation in the spinal cord of SOD1^G93A^ mice. Furthermore, we have demonstrated that verapamil reduced endoplasmic reticulum stress and suppressed glia activation in SOD1^G93A^ mice. Collectively, our study indicated that verapamil is neuroprotective in the ALS mouse model and the Ca^2+^-dependent autophagic pathway is a possible therapeutic target for the treatment of ALS.

Amyotrophic lateral sclerosis (ALS) is the most common motor neuron disease characterized by the progressive loss of motor neurons (MNs) in the spinal cord, brainstem and cerebral cortex [1]. About 10% of ALS cases are familial and among them, 20% of patients have mutations in the SOD1 (superoxide dismutase 1) gene [2]. Many studies indicated that familial and sporadic ALS shared a similar neurodegenerative pattern and were clinically and pathologically indistinguishable [3, 4], suggesting that these two forms share common molecular mechanisms in MN degeneration. The mechanisms underlying disease manifestations in ALS remain unclear, but a toxic gain of function resulting from abnormal protein aggregation is probably one of the important causes resulting in the occurrence of the disease [5]. Thus, strategies to accelerate the clearance of aggregated proteins are emerging as an attractive therapeutic target for ALS treatment.

Macroautophagy (hereafter referred to as autophagy) is a major degradation pathway that is involved in the clearance of protein aggregation and injured organelles [6]. Autophagy delivers cargo to the lysosome through sequestration into a double membrane vesicle known as autophagosomes (AVs) [7]. Then, AVs further mature and fuse to the lysosome to continue with degradation [7]. Our findings and other previous studies have demonstrated the accumulation of AVs in MNs of SOD1^G93A^ mice and ALS patients [8, 9]. Activation of autophagy might be protective in certain conditions by inducing the removal of toxic protein aggregates [10]. However, abnormalities in autophagy have been observed in numerous neurodegenerative diseases, including ALS [11]. Pharmacological and genetic modulation of autophagy could result in diverse and even opposite outcomes to the survival of ALS models [12, 13], suggesting the need for developing autophagy inducers with higher specificity and lower cytotoxicity.

Intracellular Ca^2+^ serves as a biological messenger that is involved in controlling almost all cell processes including muscle contraction, proliferation, protein synthesis and autophagy [14]. Several studies illustrated the elevation of cytosolic Ca^2+^ and the abnormalities of Ca^2+^ homeostasis in ALS MNs [15, 16]. Elevated intracellular Ca^2+^ has been shown to modulate autophagy in an mTOR-independent manner at the level of both AVs formation and AVs fusion with lysosomes [17]. Intracellular Ca^2+^ activates calpains, which are Ca^2+^-dependent cysteine proteases, that cleave several Atg proteins including Atg5 and inhibit the formation of Atg12-Atg5 [18]. A screen to identify autophagy modulators has revealed that L-type channel Ca^2+^ blockers (CCBs) act as enhancers of autophagy by increasing AVs synthesis and facilitate the clearance of autophagy substrates [19]. In SOD1^G93A^ mice, decreasing intracellular Ca^2+^ level by blocking AMPA receptors or enhancing the Ca^2+^ buffering capacity have shown protective effects [20, 21]. However, the effects of L-type CCBs on the survival of ALS MNs are still unclear.

Verapamil belongs to the dihydropyridine family of the L-type CCBs and is used clinically to treat cardiovascular disease such as high blood pressure and cardiac arrhythmias. It has been reported that verapamil causes an activation of autophagy and induction of an autophagic flux by reducing the level of intracellular Ca^2+^ [19]. The latest study indicated that verapamil could effectively transport Riluzole to brain cells which could be used to improve ALS therapy [22]. Furthermore, verapamil was found to show neuroprotective effects through novel anti-inflammatory mechanisms in *an in* vitro model of Parkinson's disease [23].

In this study, we administered verapamil to SOD1^G93A^ mice to study its effects on motor neuron survival and explore the possible mechanisms involved in ALS pathogenesis.

## MATERIALS AND METHODS

### Transgenic mice and treatment

Transgenic SOD1^G93A^ mice expressing mutant human SOD1 with a Gly93Ala substitution (B6SJL-Tg-SOD1G93A-1Gur) were originally obtained from Jackson Laboratories (No. 002726). The genotypes of the transgenic mice were identified by PCR as in our previous reports [8]. Verapamil (Sigma-Aldrich) was dissolved in ddH_2_O to a final concentration of 0.125mg/μL and further diluted with ddH_2_O before intraperitoneal injection. As there is gender differences in terms of disease progression and lifespan in SOD1^G93A^ mice [24], we used only male mice to avoid gender diversity. To assess the effect of verapamil on disease onset and survival, 24 male SOD1^G93A^ mice were randomly divided into 2 groups: (1) Tg-Vera group, intraperitoneally injected with verapamil at a dose of 25 mg/kg body weight/day (n = 12); (2) Tg-Con group, used as control, intraperitoneally injected with the same volume of ddH_2_O (n = 12). The injection was given once a day starting from 64 days after birth until the day of death. No significant side effects under this dosage were observed in our experiment.

To explore the mechanisms of neuroprotective effects provided by verapamil, 24 SOD1^G93A^ mice and 24 age- and sex-matched wild-type (WT) littermates were randomly divided into four groups: verapamil treatment at the dose of 25mg/kg body weight/day in SOD1^G93A^ mice (Tg-Vera, n = 12) or WT mice (WT-Vera, n = 12), and ddH_2_O treatment in SOD1^G93A^ mice (Tg-Con, n = 12) or WT mice (WT-Con, n = 12). These mice were sacrificed at the age of 120 days for Western blotting and skeletal muscle analysis, immunofluorescent staining, electron microscopy analysis and calpain activity assay.

All animals were housed at 21 ~ 24? and controlled 12-hour light/dark cycle. Animal use was approved by the Animal Care and Use Committee of Shanghai Jiaotong University School of Medicine and all procedures in our experiments were conducted in accordance with the guidelines of NIH for animal care.

### Behavioral Study

#### Test of motor function (Rotarod test)

Rotarod performance was examined in SOD1^G93A^ mice starting at 80 days of age. Mice were trained for 1 week prior to testing to allow them to adapt to the apparatus (Med Associates Inc., ENV-575M, USA). The rotarod test was performed every 2 days to determine the time that the mice remained on the rotating rod (4 cm diameter, 20 rpm). The date of disease onset was recorded when the mouse could not stay on the rod for 5 minutes.

#### Assessment of life span

The date of “death” was defined as the day when the mouse could not set itself upright within 30 s after being placed on a flat surface. All mice were tested every day after disease onset. All behavioral tests were performed by a technician blinded to experimental conditions throughout the entire duration of the study.

#### Immunofluorescent staining

Mice were anesthetized and perfused transcardially with phosphate buffered saline (PBS) at the age of 120 days. For histopathological analysis, the spinal cord (L4-5) was removed, post fixed and dehydrated in 15% and 30% sucrose each for 24 hours then frozen at -80 ?. For Western blot assay, the spinal cord (C1-L3) was quickly removed and preserved in liquid nitrogen for further detection. The fixed L4-5 spinal cords were serially cut at 10 μm thickness and mounted on gelatin-coated slides. The slides were incubated in 3% bovine serum in PBS containing 0.3% Triton X-100 for 1 hour at room temperature, followed by overnight incubation with primary antibodies against SMI-32 antibody (1:1000, Abcam), LC3B (1:200, CST) and SOD1 (1:200, Abcam). After thoroughly washing with PBS, the slides were incubated with Cy2-conjugated or Cy3-conjugated secondary antibodies (1:1000, Jackson Immunoresearch) then visualized at a magnification of 600 using a fluorescent microscope (Nikon, 80i).

#### Motor neuron survival analysis

A total of 200 serial sections (from L4-5) in each mouse were collected and frozen at -80 ? until further use. For Nissl staining, every 4th section was selected and to be stained with 1% Cresyl violet. The stained sections were photographed under a microscope (Olympus, IX81) and the number of MNs on both sides of the anterior horns were examined by a technician who was blinded to the experimental conditions. The number of MNs was counted as described previously [13].

For the SMI-32 positive MNs assay, sections were stained for immunofluorescence with the SMI-32 antibody according to the immunofluorescent staining method and results were captured by microscope. Twenty slices were collected per mouse at an interval of 10 slices. The number of MNs on both sides of the anterior horn were counted by investigators in a blinded manner.

#### Extraction of soluble and insoluble proteins

According to our previously reported protocol [25], the spinal cords were removed and lysed by sonication in ice-cold extraction buffer, containing 10 mM Tris-HCl, pH 8.0, 1 mM EDTA, 100 mM NaCl, 0.5% NP-40, supplemented with protease inhibitor cocktail, 1 mM PMSF and 50 mM iodoacetamide. After centrifuging at 130,000g for 15 min at 4 ?, the supernatant fraction was kept as the soluble sample. Then, the pellet as ultrasonically dissolved in resuspension buffer (10 mM Tris-HCl, pH 8.0, 1 mM EDTA, 100 mM NaCl, 0.5% NP-40, 0.5% deoxycholic acid and 2% SDS) and reserved as insoluble protein sample for further analysis by Western blotting.

#### Western blot analysis

Samples were lysed in a lysis buffer containing the following: 50 mmol/L Tris-HCl, 150 mmol/L NaCl, 1% Nonidet P40, 0.5% sodium deoxycholate, 1 mmol/L EDTA, 1 mmol/L phenylmethylsulphonyl fluoride (PMSF), with protease inhibitor cocktail (pepstatin 1 g/mL, aprotinin 1 g/mL, leupeptin 1 g/mL). Protein concentrations were measured using the BCA method and 40 μg of proteins were loaded and separated using SDS-PAGE, transferred onto polyvinylidene fluoride membranes, and blocked in 5% nonfat milk or 5% bovine serum albumin. Membranes were incubated at 4 ? overnight with the following antibodies: anti-SOD1 (1:2000, Abcam), anti-LC3 (1:1000, Sigma), anti-p62 (1:1000, CST), anti-Beclin1 (1:1000, CST), anti-mTOR (1:1000, Abcam), anti-ATG5 (1:500, MBL), anti-p62 (1:500, MBL), anti-calpain 1 (1:1000, Abcam), anti-Bip (1:1000, CST), IRE1α (1:500, CST), CHOP (1:1000, CST) and PDI (1:1000, CST), anti-cleaved-caspase-12 (1:500, CST) and anti-β-actin (1:10000, Sigma). The membranes were then incubated with appropriate peroxidase-conjugated secondary antibodies for 2 hours. Protein bands were visualized using ECL (Pierce, USA) and an image analyzer was used to quantify the densities of interested protein bands (Bio-Rad, Image lab 4.1).

#### Electron microscopy analysis

L4-5 spinal cords were fixed in 2.5% glutaraldehyde and cut into 50 μm thick sections. These sections were post fixed with 1% OsO_4_, dehydrated and embedded in Durcupan (ACM) on a microscope slide and cover-slipped. Sections were further cut by a Reichert ultramicrotome into 70 nm thick sections. The ultra-thin sections were stained with uranyl acetate and evaluated with an electron microscope.

Motor neurons were selected based on strict criteria (multipolar cells with dispersed nuclear chromatin and prominent nucleoli) including size exclusion where neurons exhibiting a maximum diameter of at least 30 μm were counted. EM images from 25 MNs per mice were captured at a magnification of 10,000 and the number of autophagosomes and autolysosomes in each captured field was measured using previously established criteria [26].

#### Pathological analysis of skeletal muscles

Biopsied gastrocnemius muscles (5 × 5 × 10 mm^3^) were dissected from the right leg and immediately in liquid nitrogen cooled isopentane. Serial cryostat sections were cut at 10 μm and stained by hematoxylin and eosin (HE) and nicotinamide adenine dinucleotide hydrogen (NADH) according to our previously described protocols [25]. Twenty-five HE images at a final magnification of 200× were counted in each mouse. The Image J software was used to measure the area of gastrocnemius muscle fibers in each group.

#### Calpain Activity Assay

A calpain activity assay kit (Abcam) was used to quantify relative calpain enzyme activity. Spinal cords of 120 days old mice were snap frozen in liquid nitrogen, and stored at -80 °C. Before the detection of calpain activity, spinal cords were pulverized using a pestle under liquid nitrogen and then ice-cold extraction buffer was added. Samples were centrifuged at 4°C at 21,000?×?g for 5?min. Super-natants were transferred to a fresh, ice-cold tube, and protein content was quantified using the Pierce BCA protein assay. Calpain activity was measured in 50 µg of total protein for each sample according to the manufacturer’s protocol. Changes in relative fluorescent units were detected at 400/505 nm and quantified with a microplate reader (BioTek, Synergy H4). Relative fluorescent units for each sample were blank-subtracted, divided by the total protein loaded, and normalized to the average of vehicle controls within each experiment. Calpain activity is reported as the percentage of WT-Con.

#### Statistics

All values were expressed as mean ± SEM. Significant differences were defined as *P <* 0.05. Disease onset and lifespan among groups were analyzed using the Kaplan-Meier survival analysis (SPSS 22.0). Other data analysis was performed using one-way ANOVA followed by Tukey’s post hoc test (Prism 7, GraphPad Inc.).

**Figure 1.**
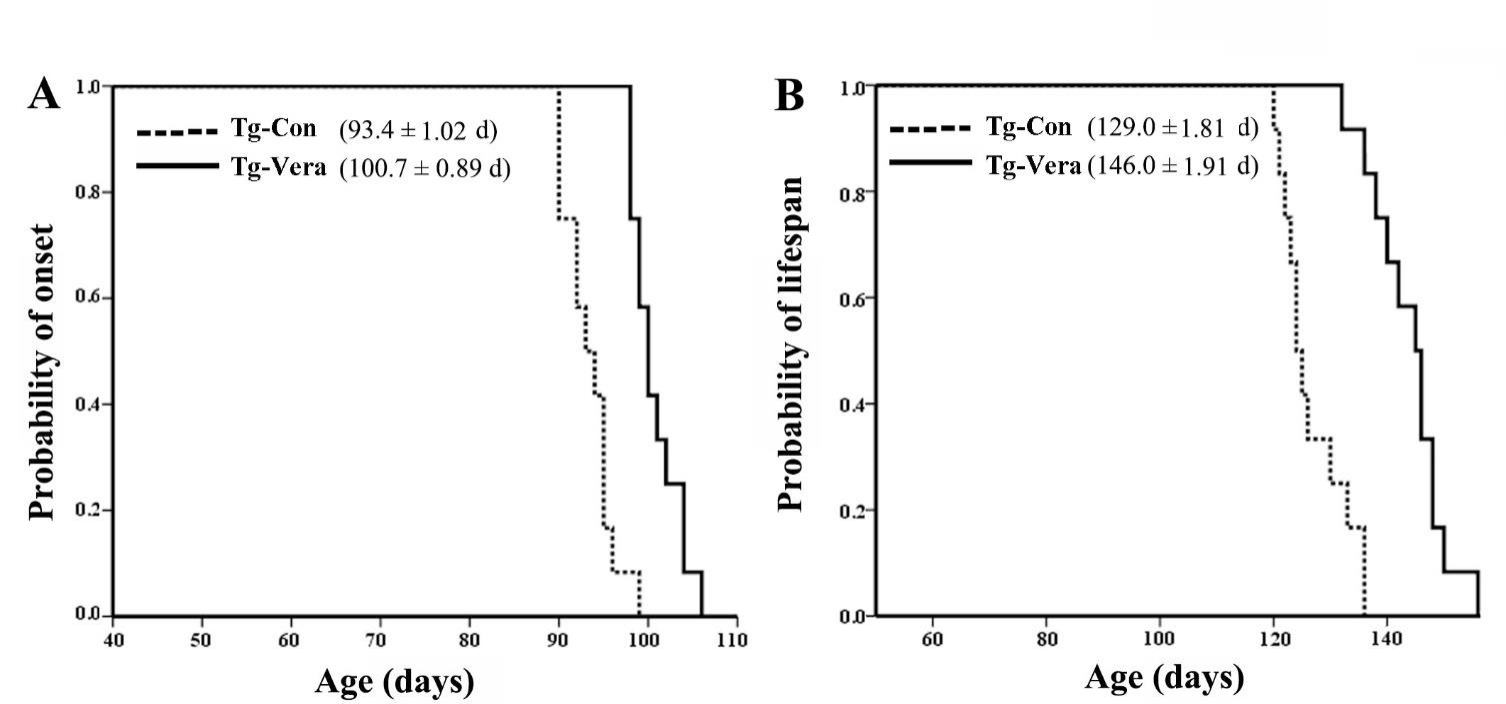
Effects of verapamil on disease onset and lifespan of SOD1^G93A^ mice. The results of Kaplan-Meier survival analysis (SPSS 22.0) showed the probability of disease onset (A) and the probability of survival (B) in Tg-Con and Tg-Vera mice, n = 12 per group. Data are presented as mean ± SEM. ^**^P < 0.01, compared with Tg-Con group.

**Figure 2.**
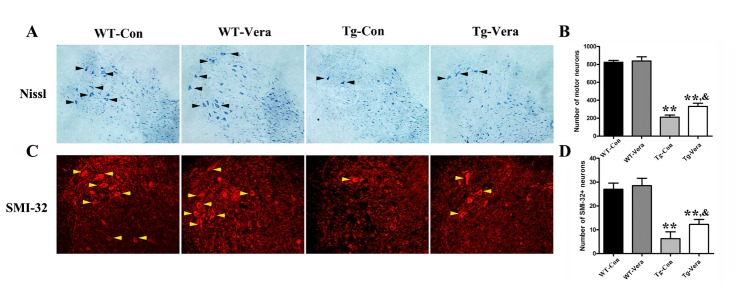
Effects of verapamil on motor neuron survival in SOD1^G93A^ mice. Representative Nissl stained photomicrographs of MNs in the anterior horn of the spinal cord (A); The number of MNs in L4-5 segments by Nissl staining (B); Representative photomicrographs of MNs in the anterior horn of the spinal cord by SMI-32 immunostaining (C). n = 3 per group; arrowheads indicate motor neurons. Scale bar = 100 μm; The mean number of SMI-32 positive MNs in both sides of one slice of an L4-5 segment (D). Data were analyzed using one-way ANOVA followed by Tukey’s post hoc test. All values are presented as mean ±SEM. ^**^P < 0.01, compared with WT-Con group; ^&^P < 0.05, compared with Tg-Con group.

## RESULTS

### Verapamil delayed disease onset and prolonged the lifespan of SOD1^G93A^ mice

The SOD1^G93A^ mice recapitulated the clinical symptoms of ALS by displaying overt hindlimb disability at 90 days, and the animals usually die around at the age of 120 days. SOD1^G93A^ mice treated with verapamil showed delay of disease onset compared with Tg-Con group mice (100.7 ± 0.89 days *vs.* 93.4 ± 1.02 days, χ2 = 16.63, *P < 0.01*) (Fig. 1A). The lifespan of SOD1^G93A^ mice treated with verapamil was 17.0 days longer than the Tg-Con group mice (146.0 ± 1.91 days *vs.* 129.0 ± 1.81 days, χ2 = 17.99, *P < 0.01*) (Fig. 1B). Moreover, there was an obvious extension in disease duration in Tg-Vera mice compared with Tg-Con mice (45.3 ± 2.63 days *vs.* 35.6 ± 2.12 days, *P < 0.01*).

**Figure 3.**
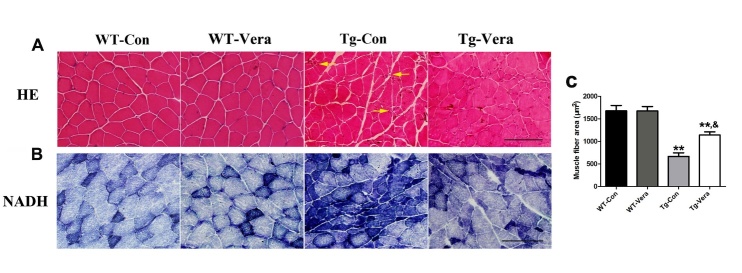
Effects of verapamil on skeletal muscle pathology in SOD1^G93A^ mice. HE (A) and NADH (B) staining of gastrocnemius muscle sections. n = 3 per group. Arrows indicate significant grouped atrophic fibers and hematoxylin inclusions. Scale bar = 100 μm. (C) Quantitative analysis of average fiber area of gastrocnemius muscle in the 4 groups. Data were analyzed using one-way ANOVA followed by Tukey’s post hoc test. All values are presented as mean ± SEM. ^**^P < 0.01, compared with WT-Con group; ^&&^P < 0.01, compared with Tg-Con group.

**Figure 4.**
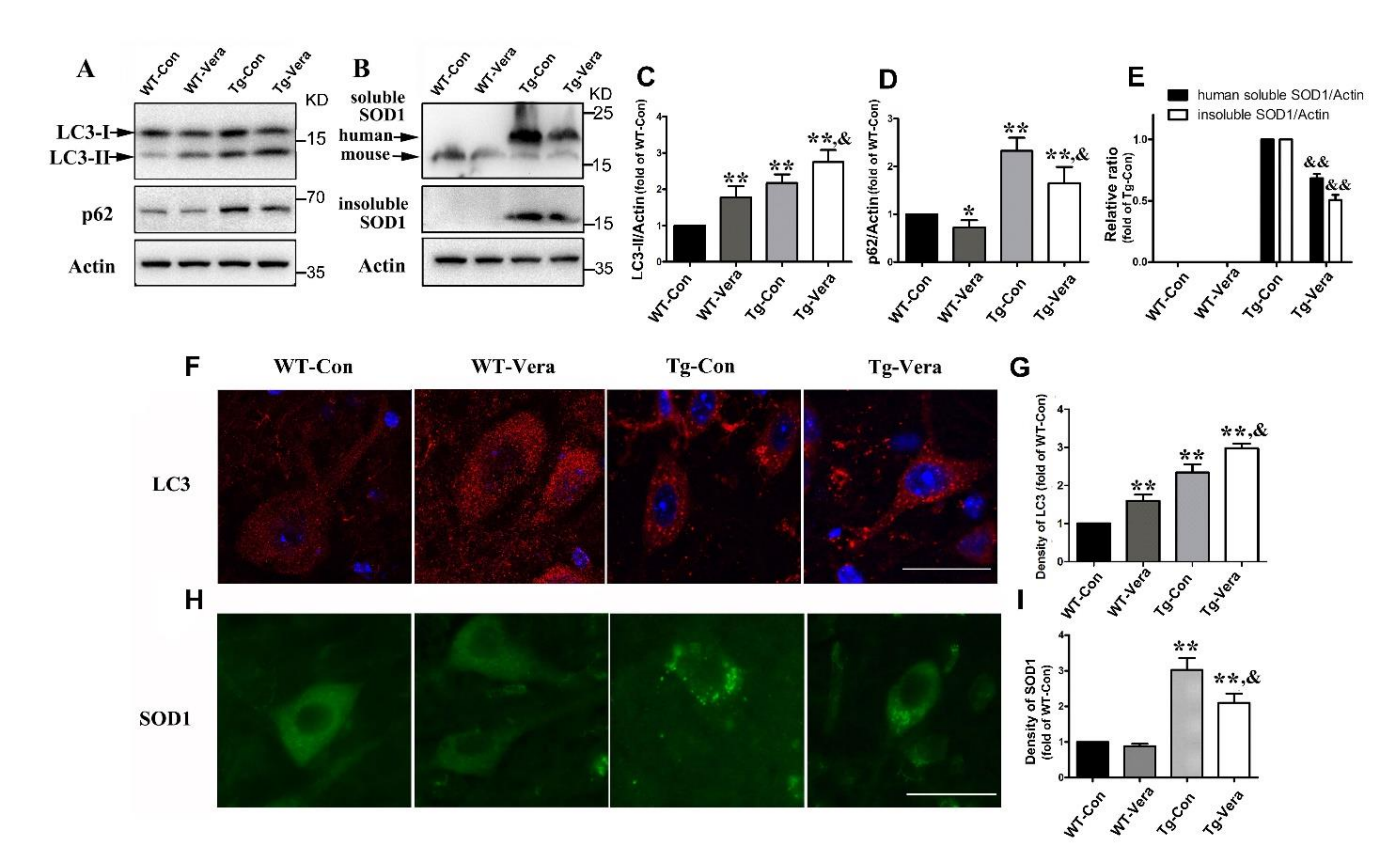
Effects of verapamil on autophagy-related proteins and protein aggregation in SOD1^G93A^ mice. Western blot analysis of protein levels of LC3 and p62 (A), soluble and insoluble SOD1 (B) in the 4 different mouse groups. Quantitative analysis of LC3B (C), p62 (D), human soluble and insoluble SOD1 protein (E) in the spinal cord of SOD1^G93A^ mice. Immunostaining of LC3 (F) and SOD1 (H) in the motor neurons of 4 mouse groups; Scale bar = 20 μm. Quantitative analysis of the density of LC3 (G) and SOD1 (I) immunofluorescence in the MNs of the 4 mouse groups. n = 3 per group. Data were analyzed using one-way ANOVA followed by Tukey’s post hoc test. All values are presented as mean ± SEM. ^**^P < 0.01 and ^*^P < 0.05, compared with WT-Con group; ^&&^P < 0.01 and ^&^P < 0.05, compared with Tg-Con group.

### Verapamil protected motor neuron survival in SOD1^G93A^ mice

To determine the effects of verapamil on MN survival, we performed Nissl staining and SMI-32 immunostaining to examine the number of MNs in the L4-5 section of the spinal cord of SOD1^G93A^ mice. According to the Nissl stain results, we found there were ~74% loss of MNs in Tg mice compared with age-matched WT mice. The number of MNs surviving in Tg-Vera mice was significantly increased when compared with Tg-Con mice (338.1 ± 17.61 *vs.* 211.0 ± 11.65, *P < 0.05*) (Fig. 2A and B). Furthermore, immunostaining with SMI-32 used to detect the survival of MNs per slice in the spinal cord of SOD1^G93A^ mice indicated that verapamil treatment could alleviate MN degeneration in SOD1^G93A^ mice (Fig. 2C). Statistical analysis showed that the number of MNs surviving in the Tg-Vera group was nearly more than twice that of the Tg-Con group (12.25 ± 1.03 *vs.* 6.52 ± 1.43, *P < 0.01*) (Fig. 2D). There was no difference in the number of MNs between WT-Vera and WT-Con mice (28.5 ± 1.55 *vs.* 27.0 ±1.29, *P > 0.05*) (Fig. 2D).

### Verapamil attenuated skeletal muscle denervation in SOD1^G93A^ mice

Besides the loss of MNs, muscle atrophy is also a ALS phenotype. To detect the therapeutic effect of verapamil in ALS, we analyzed morphological changes of the gastrocnemius muscle using HE and NADH staining. Compared to WT-Con mice, HE stains showed significant atrophic fibers, hematoxylin inclusions and central nuclei in Tg-Con mice (Fig. 3A). Verapamil treatment increased the cross-section area of muscle fibers and reduced the central nuclei in SOD1^G93A^ mice. The average muscular fiber area in Tg-Vera mice was significantly larger than that in Tg-Con mice (1141.0 ± 70.28 μm^2^ *vs.* 686.5 ± 79.69 μm^2^, *P < 0.05*) (Fig. 3C). NADH staining showed grouped type I (dark blue) and type II (light blue) muscle fibers and increased oxidative metabolism in Tg-Con mice (Fig. 3B). Verapamil treatment attenuated the grouped myofibers and reduced the oxidative muscle fibers in SOD1^G93A^ mice.

**Figure 5.**
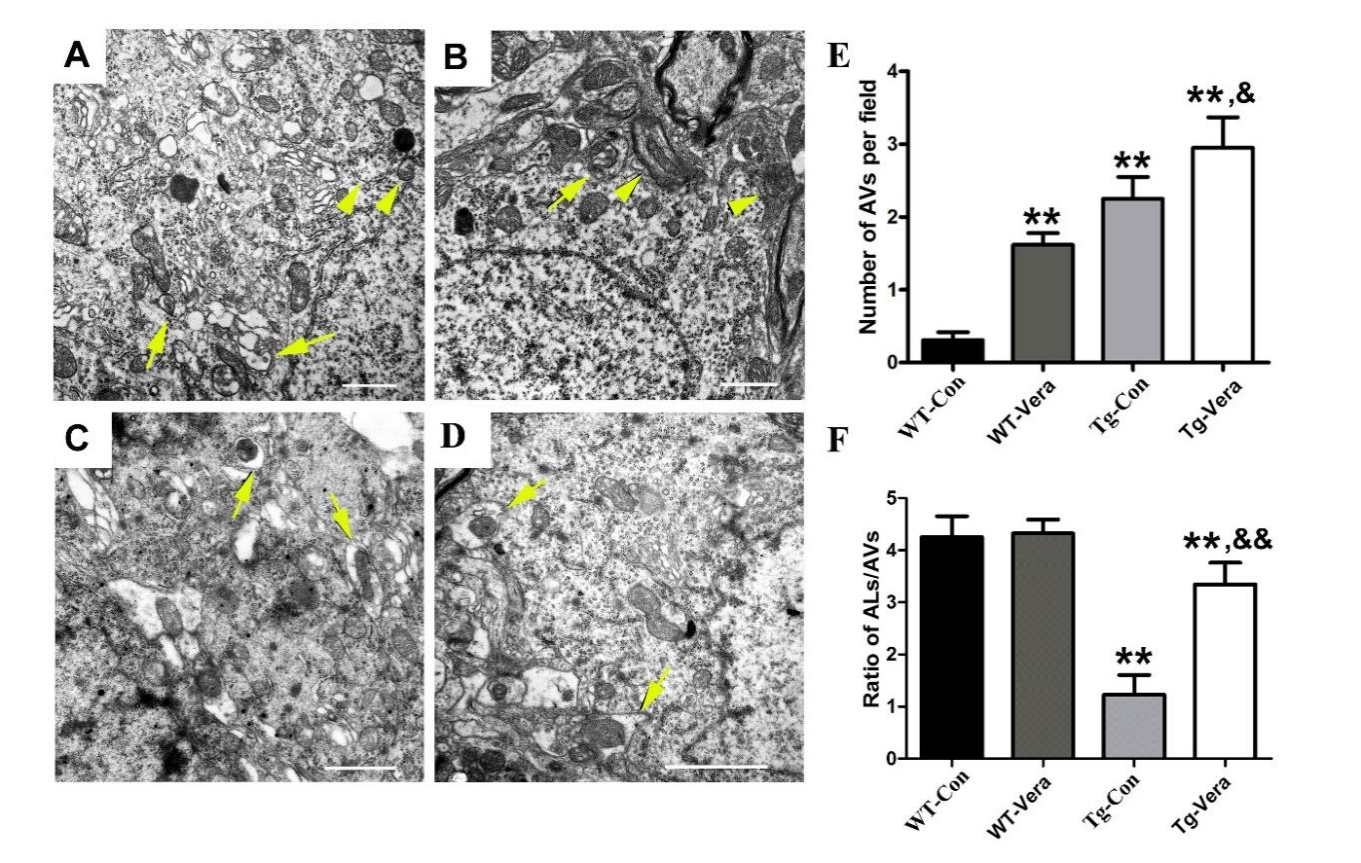
Effects of verapamil on the numbers of autophagosomes and autolysosomes in the motor neurons of SOD1^G93A^ mice. Representative EM photomicrographs of autophagosomes (marked by yellow arrowheads) and autolysosomes (marked by yellow arrows) in the motor neurons of WT-Vera mice (A) and Tg-Vera mice (B-D). (E) Quantitative analysis of the number of autophagosomes per field of 4 mouse groups. (F) Quantitative analysis of autophagosome/autolysosome ratios per motor neuron of the 4 different mouse groups. Scale bar = 1 μm; n = 3 per group. Data were analyzed using one-way ANOVA followed by Tukey’s post hoc test. All values are presented as mean ± SEM.^**^P < 0.01, compared with WT-Con group; ^&&^P < 0.01 and ^&^P < 0.05, compared with Tg-Con group.

### Verapamil induced autophagy activation and reduced SOD1 aggregation in SOD1^G93A^ mice

Abnormal SOD1 aggregation is involved in the pathogenesis of ALS and reducing mutant SOD1 protein aggregation is considered as a potential strategy to treat ALS [5]. We detected a significant decrease in SOD1 aggregates in Tg-Vera mice compared with Tg-Con mice (Fig. 4H). Statistical analysis showed that the density of SOD1 immunostaining in the MNs of Tg-Vera mice was reduced by ~30% compared with Tg-Con mice (Fig. 4I). In addition, Western blot analysis showed a 42% and 49% decrease of soluble and insoluble SOD1 protein in the spinal cords of Tg-Vera mice when compared with Tg-Con mice, respectively (both *P < 0.01*) (Fig. 4B and E).

Considering the degradation functions of autophagy in reducing abnormal aggregated proteins, we examined the expression of autophagy-related proteins such as LC3B and p62 in all groups. Quantitative analysis of Western blots showed that a 26% increase of LC3B protein in the spinal cord of Tg-Vera as compared with Tg-Con mice (*P < 0.05*) (Fig. 4A and C). Moreover, the result of immunofluorescent staining indicated a 27% increase of LC3 density in the MNs of Tg-Vera mice compared with Tg-Con mice (*P < 0.05*) (Fig. 4F and G). Meanwhile, there was a reduction in the level of p62 protein in Tg-Vera mice *vs.* Tg-Con mice (Fig. 4A). Statistical analysis of Western blots revealed a 29% reduction of p62 protein in the spinal cord of Tg-Vera *vs. Tg*-Con groups (*P <0.05*) (Fig. 4D). These results indicate that verapamil can reduce SOD1 aggregation in MNs possibly by activating autophagy in SOD1^G93A^ mice.

### Verapamil ameliorated dysfunctional autophagic flux in SOD1^G93A^ mice

The reduction of p62 in Tg-Vera mice indicated improved autophagic flux in the spinal cord of SOD1^G93A^ mice. To further determine the ultra-structure alteration in SOD1^G93A^ mice with verapamil administration, we used EM to quantify the number of autophagosomes and autolysosomes in the MNs of the different groups. EM results showed that there were double-membraned autophagosomes (marked with arrowheads) and obvious autolysosomal aggregation (marked with arrows) in the MNs of the WT-Vera (Fig. 5A) and Tg-Vera mice (Fig. 5B-D). The number of autophagosomes was increased by 31% in the MNs of Tg-Vera mice when compared with Tg-Con mice (Fig. 5E). Interestingly, we found that the size of autophagosomes in WT-vera mice seems smaller than in Tg-Vera mice or Tg-Con mice. In most of the WT-Vera autophagosomes, there were no organelles or aggregated proteins which is vastly different with Tg mice with or without verapamil treatment (Fig. 5A and B). Moreover, quantitative analysis demonstrated a significant reduction of autolysosome /autophagosome ratio in the Tg-Con mice when compared with WT-Con mice (Fig. 5F). Verapamil administration significantly improved the ratio of autolysosome /autophagosome in the MNs of SOD1^G93A^ mice (3.35 ± 0.41 *vs.* 1.23 ± 0.38, *P < 0.01*) (Fig. 5F). Taken together, EM results further indicate that the dysfunction in autophagic flux is ameliorated in verapamil-treated mice.

**Figure 6.**
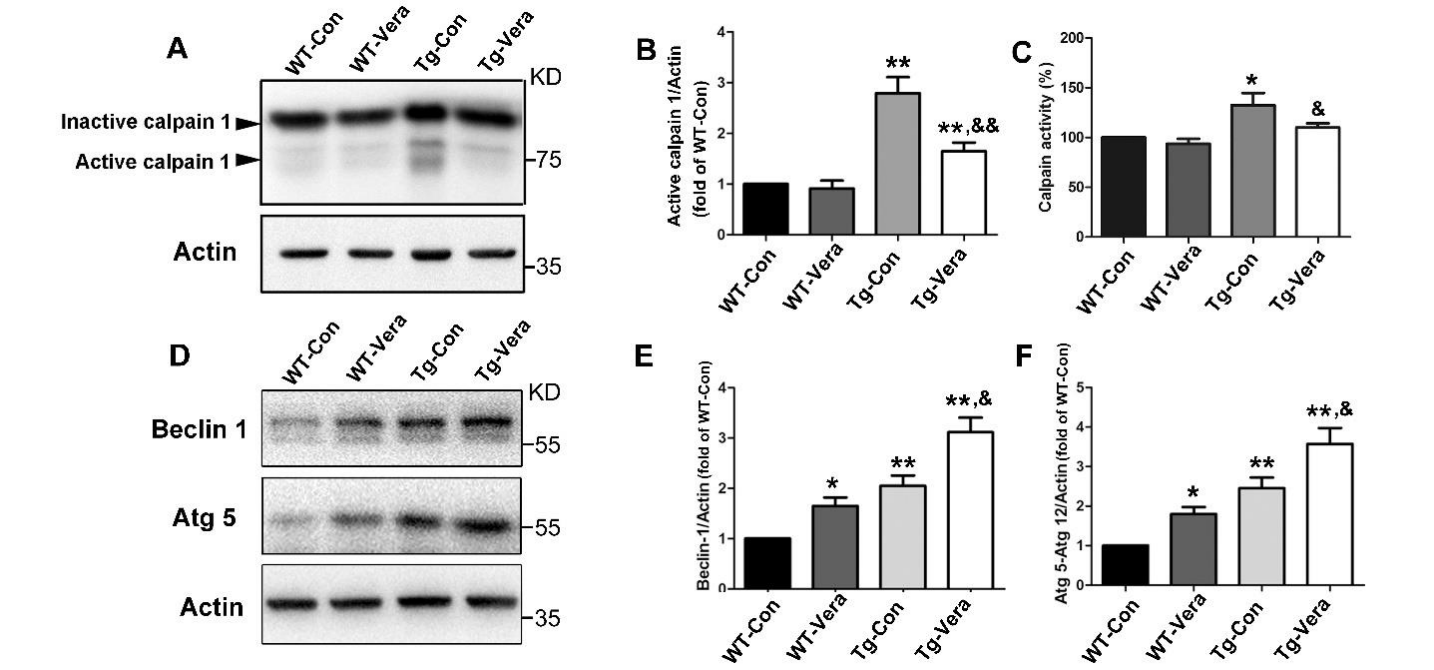
Effects of verapamil on calpain 1 and its substrate proteins. Western blot analysis of protein levels of the inactive and active calpain 1 subunit in the different groups (A). Quantitative analysis of the ratio of active calpain 1 (B) and calpain activity (C) in the different groups; (D) Western blot analysis of protein levels of Beclin-1 and Atg5. Quantitative analysis of the ratio of Beclin-1 (E) and Atg5 (F). n = 3 per group. Data were analyzed using one-way ANOVA followed by Tukey’s post hoc test. All values are presented as mean ± SEM. ^**^P < 0.01 and ^*^P < 0.05, compared with WT-Con group; ^&&^P < 0.01 and ^&^P < 0.05, compared with Tg-Con group.

### Verapamil inhibited calpain 1 expression and its pathway in SOD1^G93A^ mice

Calpain 1 is a Ca^2+^-dependent cysteine protease that plays an important role in the Ca^2+^-associated autophagy and apoptosis pathways [18]. To determine the alteration of calpain 1 related pathways, we examined the protein level of calpain 1 in the spinal cords of 4 different group mice. Western blot results indicated that the level of active calpain 1 was significantly increased in SOD1^G93A^ mice as compared with age-matched WT-Con mice, while verapamil treatment decreased the levels of calpain 1 by 41% in Tg-Con mice (Fig. 6A and B). Additionally, calpain activity was substantially decreased in the spinal cord of SOD1^G93A^ mice with verapamil treatment as compared with Tg-Con mice (Fig. 6C). Our results indicated the alteration of calpain 1 level and activity in SOD1^G93A^ mice and verapamil can reduce the calpain 1 expression in ALS mice. Moreover, compared with Tg-Con mice, verapamil administration significantly increased the levels of Beclin-1 and Atg5 in SOD1^G93A^ mice, which are the substrates for calpain 1 (Fig. 6D-F). Taken together, we speculate that verapamil may modulate autophagy associated protein expression by regulating the calpain 1 related pathway in SOD1^G93A^ mice.

### Verapamil ameliorated endoplasmic reticulum (ER) stress in SOD1^G93A^ mice

**Figure 7.**
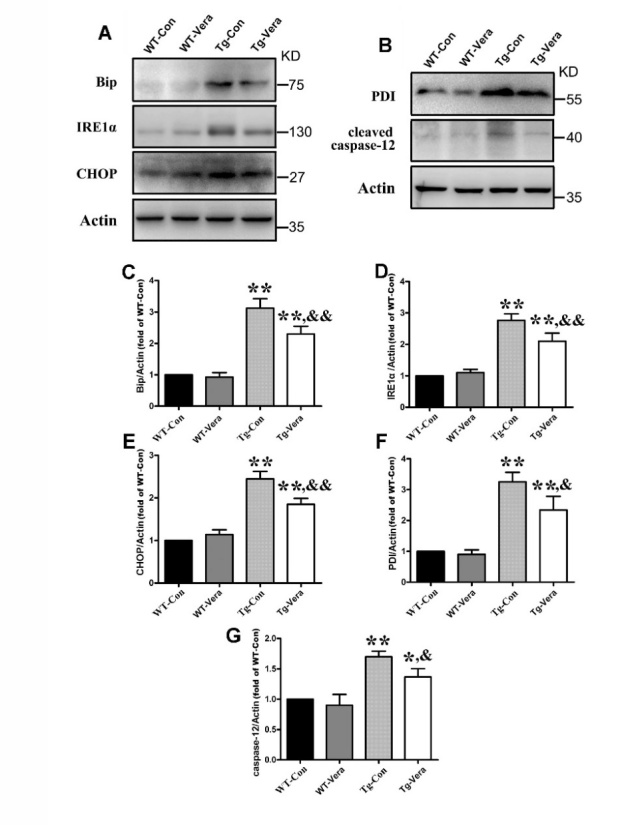
Effects of verapamil on ER stress related proteins. Western blot analysis of protein levels of (A) Bip, IRE1α, CHOP and (B) PDI, cleaved caspase-12 in the different groups. Quantitative analysis of the ratio of Bip (C), IRE1α (D), CHOP (E) and PDI (F), cleaved caspase-12 (G). n = 3 per group. Data were analyzed using one-way ANOVA followed by Tukey’s post hoc test. All values are presented as mean ± SEM.^**^P < 0.01 and ^*^P < 0.05, compared with WT-Con group; ^&&^P < 0.01 and ^&^P < 0.05, compared with Tg-Con group.

Numerous studies indicated that elevated ER stress and up-regulated ER stress proteins can result in selective neuronal vulnerability and apoptosis in ALS, which contributes to the pathogenesis of this disease [27]. To investigate the effects of verapamil on ER-related proteins, we measured the protein levels of Bip, IRE1α, CHOP, PDI and apoptosis-related protein cleaved caspase-12 in the spinal cords of all groups. Quantitative analysis of Western blot showed that Bip, IRE1α, CHOP and PDI protein levels were decreased by 26%, 23%, 24%, 26%, respectively, in Tg-Vera mice *vs.* Tg-Con mice (Fig. 7A and C-F). It was reported that ER stress leads to the activation of caspase-12, which in turn can induce apoptosis and cell death [26]. Here, we found that verapamil treatment reduced the expression of cleaved caspase-12 in SOD1^G93A^ mice (Fig. 7B and G), indicating the effects of verapamil on MN survival through the inhibition of caspase-12 induced apoptosis.

### Verapamil inhibited the activation of glial cells in SOD1^G93A^ mice

**Figure 8.**
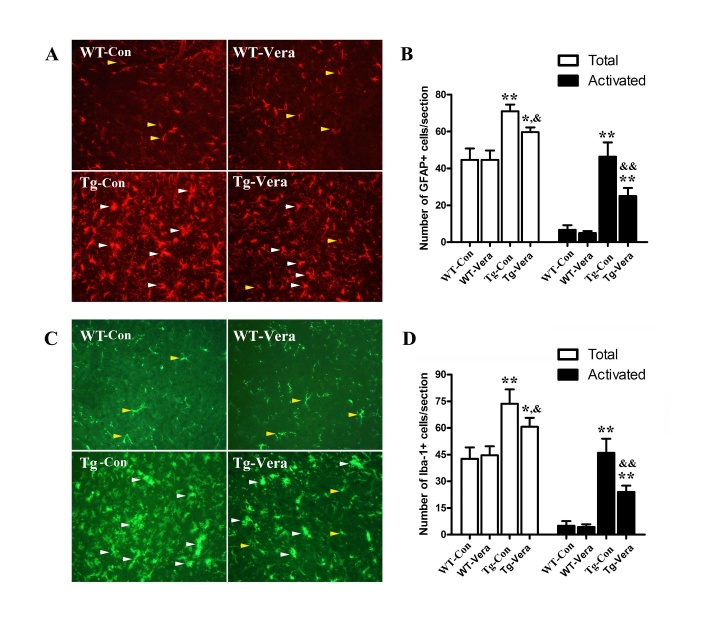
Effects of verapamil on glial activation in SOD1^G93A^ mice. Immunofluorescence labeling of GFAP (red) in the spinal cord of the 4 mouse groups (A). White arrows: typical activated astrocytes; Yellow arrows: typical resting astrocytes; Quantification of activated astrocytes in the lumbar spinal cords of each group (B); Immunofluorescent labeling of Iba-1 (green) in the spinal cord of the different groups (C). White arrows: typical activated microglia; Yellow arrows: typical resting microglia; Quantification of activated microglia in the lumbar spinal cords of each groups (D). n = 3 per group. Data were analyzed using one-way ANOVA followed by Tukey’s post hoc test. All values are presented as mean ± SEM.^**^P < 0.01 and ^*^P < 0.05, compared with WT-Con group; ^&&^P < 0.01 and ^&^P < 0.05, compared with Tg-Con group.

Glial activation in the spinal cord was investigated using immunohistochemistry. We used Iba-1 and GFAP to examine the status of microglia and astrocytes in the spinal cords of SOD1^G93A^ mice. Our results demonstrate that the resting glial cells exhibited a ramified morphology, which was characterized by small cell somata and a few long and branched processes. However, the activated astrocytes or microglia exhibited enlarged round and aggregated soma with short processes. The numbers of total astrocytes and microglia were found to be elevated at least 1-fold in the 120 days SOD1^G93A^ mice compared with the age-matched WT mice (*P < 0.01*) (Fig. 8), while the numbers of activated astrocytes and microglia in SOD1^G93A^ mice were found to be increased approximately 7- and 9-fold compared with WT mice (*P < 0.01*) (Fig. 8B and D). In contrast, mice treated with verapamil displayed a 45.8% (*P < 0.0*1) and 42.7% (*P <0.01*) reduction in the numbers of activated astrocytes and microglia, respectively (Fig. 8B and D).

## DISCUSSION

Mounting evidence support the view that abnormal protein aggregation is involved in the pathogenesis of both familial and sporadic ALS [3, 4]. Abnormalities of Ca^2+^ homeostasis contributed to the SOD1 aggregation within specific MNs [28] however, the effects of CCBs in the pathology of ALS has not yet been elucidated. Results of a screen for autophagy enhancers indicated that verapamil increased LC3B level most significantly when compared with other CCBs, which suggests verapamil’s potency as an autophagic activator [19]. In addition, as a classic drug, verapamil did not show any major toxic side effects when used in the clinical setting. In the present study, we report that verapamil could delay disease onset, prolong the lifespan and extend disease duration in the SOD1^G93A^ mouse model of ALS. Furthermore, we found that verapamil treatment could alleviate degeneration of MNs in the anterior horn of the spinal cord of SOD1^G93A^ mice, which might be related with the decreased SOD1 protein aggregation and improved autophagic flux in MNs.

Protein quality control, especially the protein degradation process, has a crucial function in neurodegenerative diseases, including ALS [29]. The presence of intracellular, insoluble inclusions composed of misfolded proteins is a hallmark of ALS pathology [30]. Our previous study reported that misfolded protein aggregation was detected in the spinal cord even at the presymptomatic stage of the disease [8, 13]. Abnormal protein accumulation has been proposed to induce MN degeneration by gain of function toxicity and perturbing multiple cellular functions including mitochondrial function, ER stress and axonal transport [31]. Therefore, clearance of mutant SOD1 toxic aggregation might show insights into the possible application of therapeutic strategies. Our findings provide evidence that verapamil was able to reduce SOD1 aggregation, particularly, by decreasing insoluble SOD1 in the MNs of SOD1^G93A^ mice. It is interesting that verapamil treatment decreased human soluble SOD1 protein in SOD1^G93A^ mice. There are two major pathways for cellular protein degradation: ubiquitin proteasome system (UPS) and autophagy. Autophagy has been shown to degrade soluble and aggregated protein substrates that are too large to enter the UPS pore, such as the toxic SOD1 protein aggregates in SOD1^G93A^ mice. UPS mainly participates in soluble SOD1 protein degradation. A previous study suggested an interconnectivity between calcium signaling and the UPS system [32]. Calcium-mediated regulation of the UPS is important in cell physiology and can be seen in primary cultured neurons [32, 33]. These studies indicate that verapamil could decrease the level of soluble SOD1 protein by influencing the UPS.

Autophagy is a main process for the degradation of aggregated proteins or damaged organelles in mammalian cells, which plays a diverse, even controversial effect for the survival of MNs in the model of ALS [34]. Clemastine, an antihistaminergic drug, modulated autophagy and neuroinflammation in NSC34 SOD1^G93A^ motor neuron cells in a time dependent manner, in parallel with the different beneficial or detrimental effects exerted by short and long treatment in SOD1^G93A^ mice [35]. A recent study reported that rilmenidine, an imidazoline-1 receptor agonist, upregulated autophagy in MNs, but worsened MN degeneration and symptom progression in SOD1^G93A^ mice, highlighting the complex effects autophagy has in ALS [36]. Using different mTOR-dependent or mTOR-independent autophagic activators, our previous study indicated the possibility of a defective autophagic flux in SOD1^G93A^ mice, specifically a dysfunction in the autophagosome-lysosome fusion process [13, 25].

Autophagy is regulated by changes in intracellular Ca^2+^ levels through an mTOR-independent pathway [19]. Abundant studies provide evidence that elevated cytosolic Ca^2+^ could induce autophagy dysfunction, which occurs at both the level of autophagosome formation and autophagosome-lysosome fusion [37]. Using EM, we demonstrated that a classic L-type Ca^2+^ channel blocker, verapamil, could not only increase the number of autophagosomes, but also significantly improve the autophagosome/lysosome ratio in MNs of SOD1^G93A^ mice. Based on these results, we speculate that verapamil can ameliorate the autophagic flux defect by inducing autophagosome formation and autophagosome-lysosome fusion. Consistent with previous reports [25], results of increased LC3B and decreased p62 levels induced by verapamil treatment further indicated its role in improving autophagic flux in SOD1^G93A^ mice.

Elevated cytosolic Ca^2+^ can activate calpains, which are a family of Ca^2+^- dependent cysteine proteases [38]. When activated, calpains influence the structure and expression of their substrate proteins by increasing enzymatic activity [38]. So far, 15 calpain members have been identified. Between them, calpain 1 and calpain 2 are ubiquitously expressed [38]. In the nervous system, calpain 1 is mostly expressed in neurons while calpain 2 is more abundant in glial cells [38]. It was reported that calpain regulated autophagy appeared to be independent of mTOR activity in that calpain inhibition induced autophagy independently of mTOR pathway [39]. Calpain-mediated hydrolysis of Beclin-1 and Atg5 under stress has been reported in nerve tissues [18, 40]. Inhibition of calpain with chemical agents or its genetic knockdown increased autophagic flux without affecting the mTOR pathway [19, 41]. Conversely, activation of calpain by elevated cytosolic Ca^2+^ inhibits autophagosome formation [19]. Studies suggest that calpain is a downstream mediator of cytosolic Ca^2+^ in the autophagic process [19, 42]. Ca^2+^ may be a key factor in modulating SOD1 toxicity in ALS MNs [28]. It was reported that Ca^2+^ specifically accumulated in the spinal cord and brain stem MNs of ALS patients as well as in SOD1^G93A^ mice [43, 44]. Studies on ALS animal models have shown that Ca^2+^ overload promoted and correlated with SOD1 aggregation [45]. Interestingly, the ER could release stored Ca^2+^ which impairs autophagosome maturation and blocks autophagosome-lysosome fusion [46]. In our study, we found elevated activated calpain 1 protein level and calpain activity in the spinal cord of SOD1^G93A^ mice, which was ameliorated by verapamil treatment. Together with previous reports [37, 39, 46], we speculate that the neuroprotective effects of verapamil may be mediated by the improvement in the calpain-induced autophagic flux and inducing autophagosomes formation and autophagosome-lysosome fusion, in SOD1^G93A^ mice.

MNs express high levels of Ca2^+^-permeable AMPA receptors which makes them more vulnerable to excitotoxicity and dysregulation of intracellular Ca2^+^ homeostasis [47, 48] due to the low calcium buffering capacity because of the lack of Ca2^+^ buffering proteins [49]. Lautenschlager *et al* reported a decelerated cytosolic Ca2^+^ clearance due to the disturbance of endoplasmic reticulum/mitochondria in MNs of SOD1^G93A^ mice, which may enhance resting Ca2^+^ levels [50]. Thus, the terminal Ca2^+^ level is likely altered in a way to enhance synaptic transmission and hyperexcitability which leads to Ca2^+^ overload induced apoptosis or necrosis in MNs of SOD1^G93A^ mice [51, 52]. More studies are needed to explore the possibility that verapamil reduces excitotoxicity and ameliorates hyperexcitability by lowering Ca2^+^ level in the MNs of SOD1^G93A^ mice.

Many studies have characterized the activation of microglia and astrocytes in the post-mortem central nervous system of ALS patients and in the spinal cord of the transgenic mouse model of ALS [53]. In ALS, microglia and astrocytes switch from a surveying state, characterized by a small cell body, to an activated state, characterized by an enlarged cell body accompanied by releasing potentially neurotoxic cytokines [53]. Our previous study showed that glia cells become activated before clinical disease onset at about 90 days of age in SOD1^G93A^ mice [13]. Of note, selective expression of mutant SOD1 in MNs did not result in a loss of motor neurons or the behavioral phenotype, which provided evidence that surrounding glial cells play an important role in MN degeneration [54]. Chemical or genetic inhibition of glia activation significantly slowed disease progression, improved survival and rescued MNs in the transgenic mouse model of ALS [55, 56]. In our study, activated Iba-1 positive microglia and GFAP positive astrocytes were highly expressed in the spinal cord of SOD1^G93A^ mice, and verapamil treatment significantly suppressed glia activation, which may partially explain the neuroprotective effects of verapamil in ALS. In the last few decades, a new gaseous neurotransmitter, hydrogen sulphide (H_2_S), has been introduced. Several studies suggested that H_2_S is mainly released by activated astrocytes and microglia, which is extremely and selectively toxic to MNs in SOD1^G93A^ mice [57, 58]. Furthermore, H_2_S can increase Ca2^+^ concentration and in turn affect the survival of MNs in SOD1^G93A^ mice [59]. More interestingly, H_2_S-induced Ca2^+^ elevation was significantly attenuated by antagonists of L-type blockers such as verapamil [60]. Further studies are warranted to investigate the effects of H_2_S in verapamil-mediated neuroprotection in SOD1^G93A^ mice.

In 1996, Miller *et al* reported that verapamil did not show significant effectiveness in slowing the clinical progression in ALS patients [61]. However, it was not a placebo-controlled, randomized, double-blinded clinical trial i.e., no Riluzole-controlled group and the study had no detailed clinical subgroup. Therefore, more clinical trials are required to confirm the effects of verapamil or other calcium channel blockers in ALS.

In conclusion, our study provides new evidence in support of L-type Ca^2+^ channels inhibitor, verapamil, for MN survival, delaying onset of ALS and prolonging lifespan in SOD1^G93A^ mice. These effects are mediated by inducing autophagy, improving autophagic flux, reducing SOD1 protein aggregation, ameliorating ER stress and inhibiting glia activation in SOD1^G93A^ mice. Our study highlights the important role of calpain 1 and its pathway in verapamil-mediated neuroprotective effects. The results from the present study indicate that cytosolic Ca^2+^ dysfunction is a critical pathological event for ALS. In fact, the elevation of calcium to toxic levels has serious implications for neuronal survival through the activation of injury mechanisms in neurodegenerative diseases such as Alzheimer's disease, Parkinson's disease and ALS [62, 63]. The approaches to reduce cellular Ca^2+^ level (such as verapamil or other CCBs) might prove to be an effective therapeutic strategy for patients with ALS and other neurodegenerative diseases.

## References

[1] Brown RH, Al-Chalabi A (2017). Amyotrophic lateral sclerosis. N Engl J Med, 377: 162-172. 28700839 10.1056/NEJMra1603471

[2] Gordon PH, Cheng B, Katz IB, Pinto M, Hays AP, Mitsumoto H, et al (2006). The natural history of primary lateral sclerosis. Neurology, 66: 647-653. 16534101 10.1212/01.wnl.0000200962.94777.71

[3] Gordon PH (2013). Amyotrophic Lateral Sclerosis: An update for 2013 Clinical Features, Pathophysiology, Management and Therapeutic Trials. Aging Dis, 4: 295-310. 24124634 10.14336/AD.2013.0400295 PMC3794725

[4] Bosco DA, Morfini G, Karabacak NM, Song Y, Gros-Louis F, Pasinelli P, et al (2010). Wild-type and mutant SOD1 share an aberrant conformation and a common pathogenic pathway in ALS. Nat Neurosci, 13: 1396-1403. 20953194 10.1038/nn.2660 PMC2967729

[5] Vidal RL, Matus S, Bargsted L, Hetz C (2014). Targeting autophagy in neurodegenerative diseases. Trends Pharmacol Sci, 35: 583-591. 25270767 10.1016/j.tips.2014.09.002

[6] Redmann M, Darley-Usmar V, Zhang J (2016). The Role of Autophagy, Mitophagy and Lysosomal Functions in Modulating Bioenergetics and Survival in the Context of Redox and Proteotoxic Damage: Implications for Neurodegenerative Diseases. Aging Dis, 7: 150-162. 27114848 10.14336/AD.2015.0820 PMC4809607

[7] Boland B, Kumar A, Lee S, Platt FM, Wegiel J, Yu WH, et al (2008). Autophagy induction and autophagosome clearance in neurons: relationship to autophagic pathology in Alzheimer’s disease. J Neurosci, 28: 6926-6937. 18596167 10.1523/JNEUROSCI.0800-08.2008 PMC2676733

[8] Li L, Zhang X, Le W (2008). Altered macroautophagy in the spinal cord of SOD1 mutant mice. Autophagy, 4: 290-293. 18196963 10.4161/auto.5524

[9] Morimoto N, Nagai M, Ohta Y, Miyazaki K, Kurata T, Morimoto M, et al (2007). Increased autophagy in transgenic mice with a G93A mutant SOD1 gene. Brain Res, 1167: 112-117. 17689501 10.1016/j.brainres.2007.06.045

[10] Cheung ZH, Ip NY (2011). Autophagy deregulation in neurodegenerative diseases - recent advances and future perspectives. J Neurochem, 118: 317-325. 21599666 10.1111/j.1471-4159.2011.07314.x

[11] Wong E, Cuervo AM (2010). Autophagy gone awry in neurodegenerative diseases. Nat Neurosci, 13: 805-811. 20581817 10.1038/nn.2575 PMC4038747

[12] Wang IF, Guo BS, Liu YC, Wu CC, Yang CH, Tsai KJ, et al (2012). Autophagy activators rescue and alleviate pathogenesis of a mouse model with proteinopathies of the TAR DNA-binding protein 43. Proc Natl Acad Sci USA, 109: 15024-15029. 22932872 10.1073/pnas.1206362109 PMC3443184

[13] Zhang X, Li L, Chen S, Yang D, Wang Y, Zhang X, et al (2011). Rapamycin treatment augments motor neuron degeneration in SOD1(G93A) mouse model of amyotrophic lateral sclerosis. Autophagy, 7: 412-425. 21193837 10.4161/auto.7.4.14541

[14] Berridge MJ, Bootman MD, Roderick HL (2003). Calcium signalling: dynamics, homeostasis and remodelling. Nat Rev Mol Cell Biol, 4: 517-529. 12838335 10.1038/nrm1155

[15] Grosskreutz J, Van Den Bosch L, Keller BU (2010). Calcium dysregulation in amyotrophic lateral sclerosis. Cell Calcium, 47: 165-174. 20116097 10.1016/j.ceca.2009.12.002

[16] Patai R, Nógrádi B, Engelhardt JI, Siklós L, et al (2017). Calcium in the pathomechanism of amyotrophic lateral sclerosis - Taking center stage? Biochem Biophy Res Commun, 483: 1031-1039. 10.1016/j.bbrc.2016.08.089 27545602

[17] He C, Klionsky DJ (2009). Regulation mechanisms and signaling pathways of autophagy. Annu Rev Genet, 43: 67-93. 19653858 10.1146/annurev-genet-102808-114910 PMC2831538

[18] Xia HG, Zhang L, Chen G, Zhang T, Liu J, Jin M, et al (2010). Control of basal autophagy by calpain 1 mediated cleavage of ATG5. Autophagy, 6: 61-66. 19901552 10.4161/auto.6.1.10326 PMC2883879

[19] Williams A, Sarkar S, Cuddon P, Ttofi EK, Saiki S, Siddiqi FH, et al (2008). Novel targets for Huntington's disease in an mTOR-independent autophagy pathway. Nat Chem Biol, 4: 295-305. 18391949 10.1038/nchembio.79 PMC2635566

[20] Tateno M, Sadakata H, Tanaka M, Itohara S, Shin RM, Miura M, et al (2004). Calcium-permeable AMPA receptors promote misfolding of mutant SOD1 protein and development of amyotrophic lateral sclerosis in a transgenic mouse model. Hum Mol Genet, 13: 2183-2196. 15294873 10.1093/hmg/ddh246

[21] Beers DR, Ho BK, Siklos L, Alexianu ME, Mosier DR, Mohamed AH, et al (2001). Parvalbumin overexpression alters immune-mediated increases in intracellular calcium, and delays disease onset in a transgenic model of familial amyotrophic lateral sclerosis. J Neurochem, 79: 499-509. 11701753 10.1046/j.1471-4159.2001.00582.x

[22] Yang T, Ferrill L, Gallant L, McGillicuddy S, Fernandes T, Schields N, et al (2018). Verapamil and riluzole cocktail liposomes overcome pharmacoresistance by inhibiting P-glycoprotein in brain endothelial and astrocyte cells: A potent approach to treat amyotrophic lateral sclerosis. Eur J Pharm Sci, 30: 30-39. 10.1016/j.ejps.2018.04.026 29704642

[23] Liu Y, Lo YC, Qian L, Crews FT, Wilson B, Chen HL, et al (2011). Verapamil protects dopaminergic neuron damage through a novel anti-inflammatory mechanism by inhibition of microglial activation. Neuropharmacology, 60: 323-380. 10.1016/j.neuropharm.2010.10.002 PMC3014428 20950631

[24] Heiman-Patterson TD, Deitch JS, Blankenhorn EP, Erwin KL, Perreault MJ, Alexander BK, et al (2005). J Neurol Sci, 236: 1-7. 16024047 10.1016/j.jns.2005.02.006

[25] Zhang X, Chen S, Song L, Tang Y, Shen Y, Jia L, et al (2014). MTOR-independent, autophagic enhancer trehalose prolongs motor neuron survival and ameliorates the autophagic flux defect in a mouse model of amyotrophic lateral sclerosis. Autophagy, 10: 1-15. 24441414 10.4161/auto.27710 PMC4091147

[26] Nixon RA, Wegiel J, Kumar A, Yu WH, Peterhoff C, Cataldo A, et al (2005). Extensive involvement of autophagy in Alzheimer disease: an immunoelectron microscopy study. J Neuropathol Exp Neurol, 64: 113-122. 15751225 10.1093/jnen/64.2.113

[27] Kikuchi H, Almer G, Yamashita S, Guégan C, Nagai M, Xu Z, et al (2006). Spinal cord endoplasmic reticulum stress associated with a microsomal accumulation of mutant superoxide dismutase-1 in an ALS model. Proc Natl Acad Sci USA, 103: 6025-6030. 16595634 10.1073/pnas.0509227103 PMC1458691

[28] Leal SS, Cardoso I, Valentine JS, Comes CM (2013). Calcium ions promote superoxide dismutase 1 (SOD1) aggregation into non fibrillar amyloid: a link to toxic effects of calcium overload in amyotrophic lateral sclerosis (ALS)? J Biol Chem, 288: 25219-25228. 23861388 10.1074/jbc.M113.470740 PMC3757185

[29] Nixon RA (2013). The role of autophagy in neurodegenerative disease. Nat Med, 19: 983-987. 23921753 10.1038/nm.3232

[30] Watanabe M, Dykes-Hoberg M, Culotta VC, Price DL, Wong PC, Rothstein JD (2001). Histological evidence of protein aggregation in mutant SOD1 transgenic mice and in amyotrophic lateral sclerosis neural tissues. Neurobiol Dis, 8: 933-941. 11741389 10.1006/nbdi.2001.0443

[31] Pasinelli P, Brown RH (2006). Molecular biology of amyotrophic lateral sclerosis: insights from genetics. Nat Rev Neurosci, 7: 710-723. 16924260 10.1038/nrn1971

[32] Mukherjee R, Das A, Chakrabarti S, Chakrabarti O (2017). Calcium dependent regulation of protein ubiquitination-interplay between E3 ligases and calcium binding proteins. Biochim Biophy Acta, 1864: 1227-1235. 10.1016/j.bbamcr.2017.03.001 28285986

[33] Laub M, Steppuhn JA, Bluggel M, Immler D, Meyer HE, Jennissen HP (1998). Modulation of calmodulin function by ubiquitin-calmodulin ligase and identification of the responsible ubiquitylation site in vertebrate calmodulin. Eur J Biochem, 255: 422-431. 9716384 10.1046/j.1432-1327.1998.2550422.x

[34] Valenzuela V, Nassif M, Hetz C (2018). Unraveling the role of motoneuron autophagy in ALS. Autophagy, 14: 733-737. 29388464 10.1080/15548627.2018.1432327 PMC5959335

[35] Perera ND, Sheean RK, Lau CL, Shin YS, Beart PM, Horne MK, et al (2018). Rilmenidine promotes MTOR-independent autophagy in the mutant SOD1 mouse model of amyotrophic lateral sclerosis without slowing disease progression. Autophagy, 14: 534-551. 28980850 10.1080/15548627.2017.1385674 PMC5915012

[36] Savina A, Paola F, Susanna A, Cinzia V (2016). Actions of the antihistaminergic clemastine on presymptomatic clemastine on presymptomatic SOD1-G93A mice ameliorate ALS disease progression. J Neuroinflammation, 13: 191. 27549088 10.1186/s12974-016-0658-8 PMC4994328

[37] East DA, Campanella M (2013). Ca2+ in quality control: an unresolved riddle critical to autophagy and mitophagy. Autophagy, 9: 1710-1719. 24121708 10.4161/auto.25367

[38] Goll DE, Thompson VF, Li H, Wei W, Cong J (2003). The calpain system. Physiol Rev, 83: 731-801. 12843408 10.1152/physrev.00029.2002

[39] Demarchi F, Bertoli C, Copetti T, Tanida I, Brancolini C, Eskelinen EL, et al (2006). Calpain is required for macroautophagy in mammalian cells. J Cell Biol, 175: 595-605. 17101693 10.1083/jcb.200601024 PMC2064596

[40] Song F, Han X, Zeng T, Zhang C, Zou C, Xie K (2012). Changes in beclin-1 and micro-calpain expression in tri-ortho-cresyl phosphate-induced delayed neuropathy. Toxicol Lett, 210: 276-284. 22366639 10.1016/j.toxlet.2012.02.011

[41] Sarkar S (2013). Regulation of autophagy by mTOR-dependent and mTOR-independent pathways: autophagy dysfunction in neurodegenerative diseases and therapeutic application of autophagy enhancers. Biochem Soc Trans, 41: 1103-1130. 24059496 10.1042/BST20130134

[42] Sato-Kusubata K, Yajima Y, Kawashima S (2000). Persistent activation of Gsα through limited proteolysis by calpain. Biochem J, 347: 733-740. 10769177 PMC1221010

[43] Kawamata H, Manfredi G (2010). Mitochondrial dysfunction and intracellular calcium dysregulation in ALS. Mech Ageing Dev, 131: 517-526. 20493207 10.1016/j.mad.2010.05.003 PMC2933290

[44] Jaiswal MK, Keller BU (2009). Cu/Zn superoxide dismutase typical for familial amyotrophic lateral sclerosis increases the vulnerability of mitochondria and perturbs Ca2+ homeostasis in SOD1G93A mice. Mol Pharmacol, 75: 478-489. 19060114 10.1124/mol.108.050831

[45] Tradewell ML, Cooper LA, Minotti S, Durham HD (2011). Calcium dysregulation, mitochondrial pathology and protein aggregation in a culture model of amyotrophic lateral sclerosis: mechanistic relationship and differential sensitivity to intervention. Neurobiol Dis, 42: 265-275. 21296666 10.1016/j.nbd.2011.01.016

[46] Hetz C, Thielen P, Matus S, Nassif M, Court F, Kiffin R, et al (2009). XBP-1 deficiency in the nervous system protects against amyotrophic lateral sclerosis by increasing autophagy. Genes Dev, 23: 2294-2306. 19762508 10.1101/gad.1830709 PMC2758741

[47] Vandenberghe W, Robberecht W, Brorson JR (2000). AMPA receptor calcium permeability, GluR2 expression, and selective motoneuron vulnerability. J Neurosci, 20: 123-132. 10627588 10.1523/JNEUROSCI.20-01-00123.2000 PMC6774105

[48] Jaiswal MK, Keller BU (2009). Cu/Zn superoxide dismutase typical for familial amyotrophic lateral sclerosis increases the vulnerability of mitochondria and perturbs Ca2+ homeostasis in SOD1G93A mice. Mol Pharmacol, 75:478-489. 19060114 10.1124/mol.108.050831

[49] Laslo P, Lipski J, Nicholson LF, Miles GB, Funk GD (2000). Calcium binding proteins in motoneurons at low and high risk for degeneration in ALS. Neuroreport, 20: 3305-3308. 10.1097/00001756-200010200-00009 11059892

[50] Lautenschlager J, Prell T, Ruhmer J, Weidemann L, Witte OW, Grosskreutz J, (2013). Overexpression of human mutated G93A SOD1 changes dynamics of the ER mitochondria calcium cycle specifically in mouse embryonic motor neurons. Exp Neurol, 247: 91-100. 23578819 10.1016/j.expneurol.2013.03.027

[51] Tadic V, Prell T, Lautenschlaeger J, Grosskreutz J (2014). The ER mitochondria calcium cycle and ER stress response as therapeutic targets in amyotrophic lateral sclerosis. Front Cell Neurosci, 8: 147. 24910594 10.3389/fncel.2014.00147 PMC4039088

[52] Jiang MC, Adimula A, Birch D, Heckman C (2017). Hyperexcitability in synaptic and firing activities of spinal motoneurons in an adult mouse model of amyotrophic lateral sclerosis. Neuroscience, 362: 33-46. 28844763 10.1016/j.neuroscience.2017.08.041 PMC5614896

[53] Philips T, Robberecht W (2011). Neuroinflammation in amyotrophic lateral sclerosis: role of glial activation in motor neuron disease. Lancet Neurol, 10: 253-263. 21349440 10.1016/S1474-4422(11)70015-1

[54] Lino MM, Schneider C, Caroni P (2002). Accumulation of SOD1 mutants in postnatal motoneurons does not cause motoneuron pathology or motoneuron disease. J Neurosci, 22: 4825-4832. 12077179 10.1523/JNEUROSCI.22-12-04825.2002 PMC6757755

[55] Boillee S, Yamanaka K, Lobsiger CS, Copeland NG, Jenkins NA, Kassiotis G, et al (2006). Onset and progression in inherited ALS determined by motor neurons and microglia. Science, 312: 1389-1392. 16741123 10.1126/science.1123511

[56] Yamanaka K, Chun SJ, Boillee S, Fujimori-Tonou N, Yamashita H, Gutmann DH, et al (2008). Astrocytes as determinants of disease progression in inherited amyotrophic lateral sclerosis. Nat Neurosci, 11: 251-253. 18246065 10.1038/nn2047 PMC3137510

[57] Davoli A, Greco V, Spalloin A, Guatteo E, Neri C, Rizzo GR, et al (2015). Evidence of hydrogen sulfide involvement in amyotrophic lateral sclerosis. Ann Neurol, 77: 697-709. 25627240 10.1002/ana.24372

[58] Lee M, Schwab C, Yu S, McGeer E, McGeer PL (2009). Astrocytes produce the antiinflammatory and neuroprotective agent hydrogen sulfide. Neurobiol Aging, 30: 1523-1534. 19631409 10.1016/j.neurobiolaging.2009.06.001

[59] Greco V, Spalloni A, Corasolla Carregari V, Pieroni L, Persichilli S, et al (2018). Proteomics and Toxicity Analysis of Spinal-Cord Primary Cultures upon Hydrogen Sulfide Treatment. Antioxidants (Basel), 7pii:E87. 10.3390/antiox7070087 PMC6070951 29996549

[60] Yong QC, Choo CH, Tan BH, Low CM, Bian JS (2010). Effect of hydrogen sulfide on intracellular calcium homeostasis in neuronal cells. Neurochem Int, 56: 508-515. 20026367 10.1016/j.neuint.2009.12.011

[61] Miller RG, Smith SA, Murphy JR (1996). A clinical trial of verapamil in amyotrophic lateral sclerosis. Muscle Nerve, 19: 511-515. 8622731 10.1002/mus.880190405

[62] Fairless R, Williams SK, Diem R (2014). Dysfunction of neuronal calcium signalling in neuroinflammation and neurodegeneration. Cell Tissue Res, 357: 455-462. 24326615 10.1007/s00441-013-1758-8

[63] Popović M, Popović N, Jovanova-Nesić K, Bokonjić D, Dobrić S, Kostić VS, et al (1997). Effect of physostigmine and verapamil on active avoidance in an experimental model of Alzheimer's disease. Int J Neurosci, 90: 87-97. 9285290 10.3109/00207459709000628

